# Mesenchymal Stem Cell-Derived Extracellular Vesicles in Cancer Therapy Resistance: from Biology to Clinical Opportunity

**DOI:** 10.7150/ijbs.88500

**Published:** 2024-01-01

**Authors:** Chan Shan, Yan Liang, Kun Wang, Peifeng Li

**Affiliations:** 1 Institute of Translational Medicine, The Affiliated Hospital of Qingdao University, College of Medicine, Qingdao University, Qingdao 266021, China.; 2 Department of Pharmaceutics, School of Pharmacy, Qingdao University, Qingdao 266021, China.

**Keywords:** mesenchymal stem cell, extracellular vesicle, cancer, therapy resistance

## Abstract

Mesenchymal stem cells (MSCs) are a type of stromal cells characterized by their properties of self-renewal and multi-lineage differentiation, which make them prominent in regenerative medicine. MSCs have shown significant potential for the treatment of various diseases, primarily through the paracrine effects mediated by soluble factors, specifically extracellular vesicles (EVs). MSC-EVs play a crucial role in intercellular communication by transferring various bioactive substances, including proteins, RNA, DNA, and lipids, highlighting the contribution of MSC-EVs in regulating cancer development and progression. Remarkably, increasing evidence indicates the association between MSC-EVs and resistance to various types of cancer treatments, including radiotherapy, chemotherapy, targeted therapy, immunotherapy, and endocrinotherapy. In this review, we provide an overview of the recent advancements in the biogenesis, isolation, and characterization of MSC-EVs, with an emphasis on their functions in cancer therapy resistance. The clinical applications and future prospects of MSC-EVs for mitigating cancer therapy resistance and enhancing drug delivery are also discussed. Elucidating the role and mechanism of MSC-EVs in the development of treatment resistance in cancer, as well as evaluating the clinical significance of MSC-EVs, is crucial for advancing our understanding of tumor biology. Meanwhile, inform the development of effective treatment strategies for cancer patients in the future.

## Introduction

Cancer is a major global health concern, causing a significant number of illnesses and deaths. As people get older, the number of cancer cases will keep rising, and patients may be diagnosed at a younger age^1^. Despite the many cancer treatments available for clinical use, including surgery, radiation, chemotherapy, targeted therapy, and immunotherapy, resistance to therapy is common and can lead to poor outcomes^2^. To date, several mechanisms have been identified to be associated with cancer therapy resistance, such as reducing drug uptake, increasing efflux mediated by transporters, decreasing apoptosis, altering autophagy, changing cell cycle, enhancing DNA damage repair, regulation by noncoding RNAs (ncRNAs), increasing platelet aggregation, and modifying drug metabolism^2^. However, the exact mechanism of resistance to cancer treatment has not been fully developed. The identification of regulatory agents and factors in the tumor microenvironment (TME) can shed light on the causes and molecular mechanisms underlying cancer therapy resistance. Targeting or intervening in these media could help alleviate or even reverse resistance to cancer therapies, leading to improved clinical outcomes.

Mesenchymal stem cells (MSCs), also known as "mesenchymal stromal cells", are a group of cells with self-renewal and multi- lineage differentiation^3^. MSCs can be obtained from various human tissues and compositions, including bone marrow, adipose tissue, dental pulp, umbilical cord, menstrual blood and amniotic membrane. Depending on their pluripotency, MSCs have the potential to differentiate into many cell types, including osteocytes, chondrocytes, adipocytes, myocytes, and more, making them valuable tools in regenerative medicine. Numerous studies have confirmed that MSCs are important components of the TME and associated with cancer progression^4^. As a crucial player in TME reprogramming, MSCs play a role in and manage communication with neighboring cells, transmitting essential cellular information that impacts the growth, differentiation, and function of other cells. MSCs have the ability to influence the actions of immune cells^5^. This includes assisting in the transition of macrophages to the M2 type, hindering neutrophil function, and guiding differentiation and proliferation of B and T lymphocytes. On the flip side, MSCs act as a framework for the growth of tumor cell and control their growth through the secretion of various substances, including cytokines, chemokines, and growth factors^6^. Recent studies have shown that, alongside their effects on TME reprogramming through soluble secretions, MSCs facilitate cell signaling between themselves and neighboring receptor cells through bioactive particles.

In the past few decades, extracellular vesicles (EVs) have been identified as a means of intercellular communication in recent years and have gained growing interest^7^. EVs have been found to carry a variety of bioactive substances, including DNA, RNA, functional proteins, lipids, and metabolites, which play a crucial role in communication between different cells and tissues, thereby regulating a variety of physiological and pathological processes, including cancer. Notably, an increasing number of studies have shown that EVs secreted by MSCs (MSC-EVs) may play a crucial role in regulating cancer therapy resistance by mediating material exchange and signal transduction with cancer cells and other stromal cell^8^. In addition, due to their accessibility, stability, safety, biocompatibility, and other beneficial properties, MSC-EVs are anticipated to have clinical applications for drug delivery and targeted treatment to improve outcomes for cancer patients^9^, ^10^.

In the following sections, we summarized the basics of biosynthesis, characteristics, and identification of MSC-EVs, focusing on the importance of MSC-EVs and their cargoes in cancer therapy resistance. At the same time, we also explore the potential role of MSC-EVs in clinical cancer therapy, which could introduce novel concepts for precise and effective cancer treatment.

## Basics of MSC-EVs

### Biogenesis of EVs

**Figure 1:**
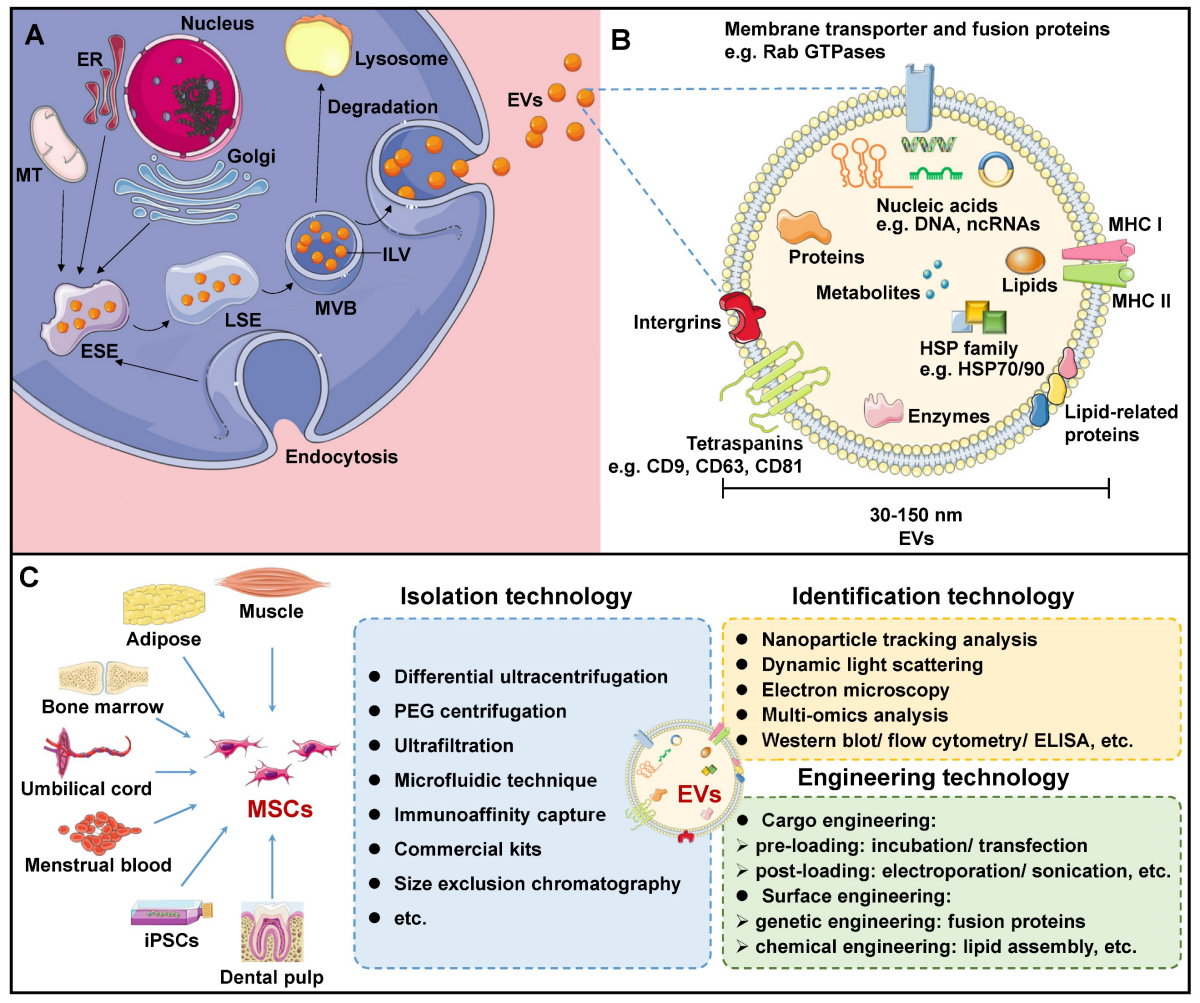
Overview of mesenchymal stem cell-derived extracellular vesicles (MSC-EVs). A. Biogenesis of EVs. B. Biomarkers and contents of EVs. C. MSC-EV sourcing, isolation, identification, and engineering technologies. ELISA: Enzyme-linked immunosorbent assay; ER: Endoplasmic reticulum; ESE: Early-sorting endosome; EV: Extracellular vesicle; HSP: Heat shock protein; ILV: Intraluminal vesicle; LSE: Late-sorting endosome; MHC: Major histocompatibility complex; MT: Mitochondrion; MSC: Mesenchymal stem cell; MVB: Multi-bubble body; PEG: Polyethylene glycol.

EVs are lipid structures ranging in size of 30-150 nm, which transport various cellular components such as DNA, RNA, lipids, metabolites, as well as cytoplasmic and cell surface proteins^7^. Over the past few decades, EVs have been detected in almost all bodily fluids, including blood, urine, milk, ascites, saliva, amniotic fluid, and even sweat. The biogenesis of EVs initiates in the cellular plasma membrane. In some situations, the cell membrane invaginates and encloses certain proteins and molecules located on the cell surface through the endocytosis process to create early-sorting endosomes (ESEs)^11^. ESEs then gradually develop into late-sorting endosomes (LSEs), and further invaginate to produce multi-bubble bodies (MVBs) that contain intraluminal vesicles (ILVs). MVBs then fuse with lysosomes for degradation, or with plasma membranes to release ILVs, thus forming EVs upon exiting the cell (Figure 1A).

Many proteins are involved in EV membrane formation and cargo loading. Endosomal sorting complexes required for transport (ESCRT) have been demonstrated to be critical regulators of ILV formation^11^, ^12^. The ESCRT complex consists of four ESCRT-0, I, II, III subcomplexes that recognize ubiquitin-modified membrane proteins. Among them, ESCRT-0 is responsible for capturing polyubiquitinated cargoes. ESCRT-0 recruited ESCRT-I and delivered cargo to it. ESCRT-I, in turn, recruited ESCRT-II to co-induce endosome membrane invagination. ESCRT-III is responsible for severing the neck of the budding membrane, leading to the formation of MVBs. In addition, apoptosis-linked protein 2-interacting protein X (ALIX), vesicle trafficking protein 1, vacuolar protein sorting-associated protein 4, and tumor susceptibility gene 101 (TSG101) support the ESCRT complex in EV biogenesis^13^. It should be noted that an alternative ESCRT-independent pathway mediates EV biogenesis^14^. In this pathway, heat shock proteins (HSPs) - including HSP60, HSP70, and HSP90 - as well as members of the tetraspanin family - such as CD63, CD81, CD82, CD37, and CD9 - play essential roles in membrane formation and cargo sorting^7^.

Once formed, MVBs are driven to and fused with the plasma membrane, and further released into the extracellular region to form EVs. Studies have verified the involvement of small GTPase proteins in the RAB family in EV secretion. In general, Rab27a is closely related to the size of MVBs, while Rab27b affects the intracellular distribution of MVBs and their fusion with plasma membranes^15^. After being secreted, EVs specifically recognize and interact with the surface protein of the recipient cells, or enter the recipient cells directly through endocytosis, resulting in EV internalization, thereby performing their role as a communication medium^16^, ^17^. Although functional proteins and underlying mechanisms involved in EVs biogenesis have been extensively described, the rate-limiting steps and regulatory pathways still require further elucidation.

### Content of MSC-EVs

The components of EVs vary according to the original cell and formation pathway, determining their function upon entering the recipient cells. Certain components are shared among all EVs, while some are specific to tissues and cells. Generally, EV transport lipids, functional proteins, DNAs, RNAs, and more^18^, ^19^. EVs are mainly composed of membrane lipids, such as cholesterol, sphingomyelin, glycosphingolipids, and phosphatidylserine, alongside small amounts of other lipids that are sequestered from the cytoplasm during ILV formation^20^. After EVs are released from the cell, lipids in the lipid bilayer maintain the same asymmetrical distribution as in the plasma membrane of the parent cell^21^. Specifically, sphingolipids and phosphatidylcholine are present in the outer membrane, while other lipids reside in the inner membrane. Based on the compositional analysis of lipids in EVs, valuable insights into the origin of EVs can be obtained. Additionally, upon fusion with EVs, the lipid composition of the recipient cells undergoes alterations, disrupting its cellular homeostasis and influencing its behavior^20^.

EV contains a wealth of functional proteins associated with biogenesis, structural stability, and signal transduction, including RAB GTPases, the ESCRT complex, ALIX, and TSG101^19^, ^22^. In addition, during the sorting of EV cargoes, some proteins that are evolutionarily conserved are encapsulated within EVs. These include tetraspanins (e.g. CD9, CD63, CD81, CD82), members of the HSP family (e.g. HSP60, HSP70, HSP90), integrin, major histocompatibility complex (MHC) I and II, among others (Figure 1B). Specifically, MSC-specific proteins, such as CD44, CD45, CD73, CD90, and CD105, are expressed in MSC-EVs, which can be employed for EV traceability^23^. Based on these MSC-specific biomarkers, Roccaro et al. identified distinct protein components in bone marrow mesenchymal stem cell (BMSC)-derived EVs and compared to BMSCs, suggesting selective encapsulation and transport of certain proteins by EVs to recipient cells^24^. To gain a more thorough understanding of protein information in MSC-EV, proteomic techniques are necessary. Kim et al. identified 730 proteins from BMSC-EVs, including MSC surface markers (such as CD9, CD63, CD81, CD109, etc.), surface receptors, and signaling molecules that facilitate cell proliferation, adhesion, migration, and morphogenesis^25^.

In addition, RNAs, especially ncRNAs, are a class of important regulatory content of EVs. MicroRNAs (miRNAs) is one of the most well studied EV RNAs. Research has demonstrated that miRNAs are specifically packaged into EVs rather than at random. Based on current research progress, several signaling pathways, such as neutral sphingomyelinase 2-dependent pathway, heterogeneous nuclear ribonucleoproteins family-dependent pathway, the 3′ end of miRNA dependent pathway, and the RNA-induced silencing complex-dependent pathway have been demonstrated to be involved in the selective sorting of miRNAs into EVs^26^-^29^. A growing number of studies have demonstrated that MSC-EVs possess the ability to selectively transport miRNAs and impact the behavior of neighboring cells, particularly cancer cells. For example, several miRNAs present in BMSC-EVs can be internalized by adjacent lung cancer cells, thereby activating STAT3-mediated epithelial-mesenchymal transitions (EMT) and promoting invasion and metastasis in lung cancer^30^. In another separate study, human umbilical cord MSC (UC-MSC)-EV deliver miRNA-375 was demonstrated to possess the capability to inhibit esophageal cancer occurrence and progression^31^.

The most stable EV cargoes are DNAs. EV DNAs are primarily composed of single-stranded DNAs, double-stranded DNAs and mitochondrial DNAs from parent cells, potentially resulting from normal DNA metabolism or DNA damage under specific conditions^32^-^34^. These DNA fragments may contain cancer-specific mutations, which are thought to be a response to oxidative stress and energy deficiencies that occur during cancer initiation and progression. That is, the cell releases DNA fragments accumulated within it through EVs. Particularly, EVs released by BMSCs are capable of transferring DNA fragments into recipient cells, where it can integrate into the host genome^35^. Moreover, EV DNAs have been found to play a role in the immune response and can affect cancer development through dendritic cells (DCs) activation and the release of inflammatory factors by neutrophils^36^. In sum, EVs contain diverse bioactive components that promote intercellular communication and have significant involvement in numerous physiological and pathological mechanisms. However, the mechanisms about DNA fragments capsulation and secretion from parent cells into EVs remains unclear and are worthy to be studied further.

### Isolation of EVs

Due to the critical potential of EVs in biological investigation and clinical use, there is an immediate need to acquire a substantial amount of pure EVs. Multiple techniques for EV isolation have been employed, including centrifugation, polymer precipitation, size-dependent isolation, immunoaffinity capture, and microfluidic techniques (Figure 1C).

Centrifugation technology is widely recognized as the gold standard for EV separation due to its ease of use and high efficiency^37^. This technology necessitates the application of various centrifugal forces at different rotational speeds. However, this method is time-intensive and yields a low level of EV purity. Polyethylene glycol (PEG) is frequently utilized in polymer precipitation techniques to displace water molecules, leading to a reduction in solubility and subsequent precipitation of EV particles^38^. In recent times, a number of EV extraction kits utilizing PEG precipitation have been commercialized. Polymer precipitation enhances the purity of EVs, but inevitably leads to the introduction of polymer impurities.

Size-dependent EV separation technology mainly includes ultrafiltration and size exclusion chromatography (SEC). Ultrafiltration uses membranes of specified pore diameters to eliminate proteins and nucleic acids that are smaller than desired EV sizes, producing a purer EV without requiring special equipment or reagents^39^. SEC is a technique for separating substances with different molecular weights based on their elution times within the chromatographic column^40^. Biological molecules such as proteins and nucleic acids may enter the pores of the stationary phase in the chromatographic column and elute at a later time, whereas EV has a shorter evaporation time. However, SEC is not highly specific to EV subtypes of varying sizes.

Immunocapture is an efficient technique for EV isolation that exploits the interactions between EV surface proteins and antibodies or ligands that are immobilized on magnetic beads^41^. This technique can selectively isolate specific EV subtypes based on their surface markers such as CD9, CD63, and CD81. However, immunophilic capture is not suitable for large sample volumes and is limited to cell-free specimens. In addition, tumor heterogeneity may impair immune recognition mechanisms.

Microfluidic technology has emerged as an innovative method for the separation of EVs by leveraging their biological, physical, and chemical properties such as size, density, and immunoreactivity^42^. In recent years, this technology has demonstrated remarkable efficacy in EV separation. Notably, microfluidic technology offers advantages such as the ability to handle small sample sizes, high sensitivity, rapid detection, high throughput, and cost-effective detection. Furthermore, it can be seamlessly integrated with conventional EV extraction techniques and combined with acoustic wave and microfluidic viscoelasticity approaches^43^, ^44^. Taken together, efficient and cost-effective isolation of numerous pure EVs is a vital requirement for EV research, requiring the development of new and dependable EV separation techniques.

### Identification of MSC-EVs

EVs play a crucial role in both basic and clinical studies. Therefore, accurate identification and strict quality control of EVs are of paramount importance. Electron microscopy (EM), dynamic light scattering (DLS), and nanoparticle tracking analysis (NTA) are established methodologies that can be employed to evaluate the physical and chemical attributes of EVs, encompassing parameters such as particle size and size distribution (Figure 1C). EM is a critical imaging technique used to analyze the microstructures of biological samples, specifically EVs^45^. Under an EM, an EV appears as a cup-shaped structure measuring 30-150nm in diameter with a distinct membrane. However, dehydration and fixation during EM sample preparation may alter the morphology of EVs^46^. In addition, DLS is a method used to measure the size distribution and concentration of particles in Brownian motion by detecting fluctuations in light scattering intensity^47^. DLS is commonly employed to measure EV dimensions and distributions. However, DLS has specific requirements for uniformity in EV size, as larger particles may interfere with fluctuations in the light intensity signal of smaller particles. Moreover, NTA is a novel method for direct and real-time measurement of EV particle size and size distribution^48^. Because NTA can detect the scattered light signal from each particle, it allows accurate determination of EVs in complex samples. However, as it relies on monitoring optical signals, the accuracy and resolution of measurements remain somewhat inconsistent.

When characterizing EV components, omics analysis is required. RNAs are critical component of EVs. It is demonstrated that ncRNAs are the primary RNA species enriched in EVs, comprising miRNAs, circular RNAs (circRNAs), long ncRNAs (lncRNAs), etc^7^. NcRNAs present in MSC-EVs have been demonstrated to have a pivotal regulatory function in the pathogenesis of various types of cancer. For instance, miRNAs carried by MSC-EVs have been proven to be a potential therapeutic strategy of beast cancer, hepatocellular carcinoma (HCC), ovarian cancer, pancreatic cancer and so on^49^-^52^. In the recent study of Yao et al., BMSC-EV circ_0030167 was demonstrated to inhibit the proliferation, migration, and invasion in cancer cells through targeting the miR-338-5p/wif1/Wnt 8/β-catenin signaling, presenting potential targets for the intervention of pancreatic cancer^53^. In another separate research, LINC00461 was found to be secreted by MSCs through EVs, further promoting the proliferation of multiple myeloma cells^54^. Protein is the direct performer of vital movement. Numerous studies have verified that EVs are enriched with abundant functional proteins, which can be classified into three groups^55^. The first category comprises of glycosylphosphatidylinositol anchored proteins and transmembrane proteins from the cell membrane and endosome, providing evidence of the lipid bilayer structure of EVs. Regarding MSC-EVs, they not only share typical EV surface biomarkers, but also display surface markers specific to MSCs, which can be utilized for MSC-EV identification^23^. For example, BMSCs were identified as CD90 and CD29 positive and CD45 negative^56^.The second category consists of cytoplasmic proteins, including membrane binding proteins and cytoplasmic domains of transmembrane proteins. Besides, this subset also includes cytoskeletal proteins and cytoplasmic enzymes. The third classification refers to additional proteins incorporated during the EV purification process. These are non-EV constituents that can be utilized to evaluate whether the purity of EVs meets the necessary standards. For example, during the isolation of EVs from plasma, apolipoprotein A1/2, apolipoprotein B, and albumin are mixed in the sample, which can serve as negative indicators to evaluate the purity of EV samples^57^. Additionally, western blotting, enzyme-linked immunosorbent assay, and flow cytometry can be utilized to qualitatively and quantitatively detection of the proteins markers for EV identification. In total, the methodology for isolating and identifying EVs needs to be enhanced to utilize them as a novel therapeutic approach for diseases.

## MSC-EVs Regulate Cancer Therapy Resistance

The most significant barrier to clinical cancer treatment is therapy resistance, which is closely linked to increased rates of recurrence and mortality. Therefore, it is crucial to understand the underlying mechanisms of therapeutic resistance in order to develop strategies for effectively eliminating cancer and preventing tumor relapse. Currently, cancer therapy resistance is commonly attributed to the following mechanisms: the rare pre-existing drug-resistant clones induce cancer relapse and therapy ineffectiveness; the adaptive response of cancer cells to treatment involves phenotypic plasticity (e.g. dedifferentiation, transdifferentiation, and altered metabolism) and changes in the TME (e.g. activation of cancer stem cells); drug-related mechanisms (such as enhanced efflux, drug inactivation, or alterations to drug targets)^58^-^61^. Novel concepts are required to uncover the fundamental mechanisms of resistance to cancer therapy. Based on this, innovative strategies are proposed to monitor and combat resistance in order to enhance the clinical effectiveness of cancer treatment. The significant role of MSC-EVs in cancer development, progression, and therapy has been well-established and is expected to emerge as a promising anticancer modality for clinical implementation. In this section, we highlight the advancements of MSC-EVs in resistance to cancer therapy, including radiotherapy, chemotherapy, targeted therapy, immunotherapy, and endocrine therapy.

### MSC-EVs in radiotherapy resistance

**Figure 2:**
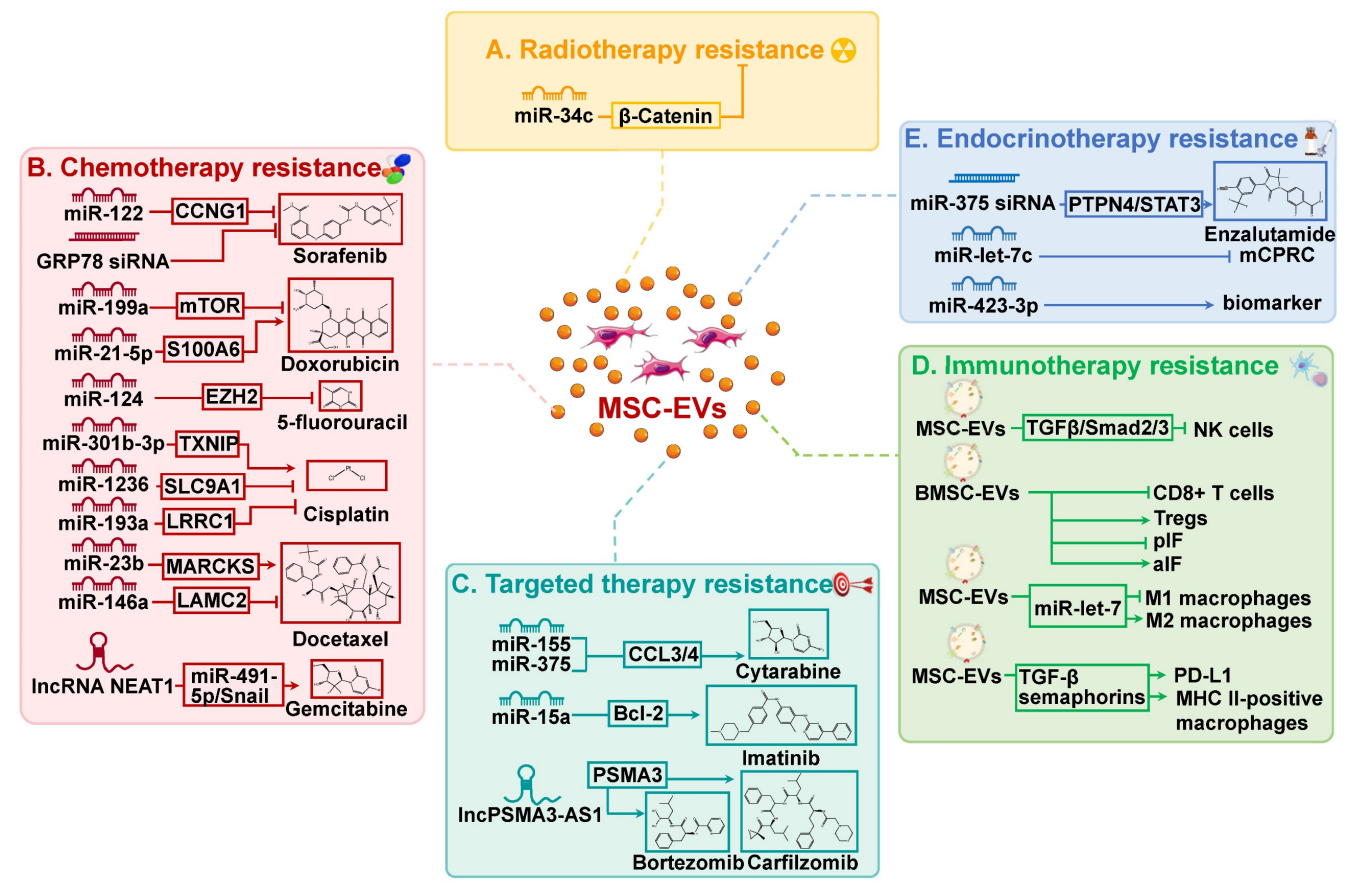
Mesenchymal stem cell-derived extracellular vesicles (MSC-EVs) play important roles in resistance to cancer therapies, including radiotherapy (A), chemotherapy (B), targeted therapy (C), immunotherapy (D), and endocrinotherapy (E). aIF: Anti-inflammatory cytokines; BMSC: Bone marrow mesenchymal stem cell; EV: Extracellular vesicle; lncRNA: Long non-coding RNA; mCPRC: Metastatic castration-resistant prostate cancer; MHC: Major histocompatibility complex; miR: MicroRNA; MSC: Mesenchymal stem cell; PD-L1: Programmed cell death protein-1 ligand 1; pIF: Pro-inflammatory cytokines; Treg: Regulatory T cell.

Radiotherapy is commonly utilized in clinical settings, with approximately half of all patients undergoing this treatment^62^. Radiotherapy can be used as a standalone treatment or in conjunction with other therapies, such as surgery and chemotherapy, to achieve improved outcomes. Despite the intrinsic or acquired resistance of cancer cells to radiotherapy, the re-growth of cancer cells and cancer recurrence frequently occur. Radiation has been observed to affect EV secretion by parent cells, EV uptake by recipient cells, and EV composition^63^. EVs, in turn, facilitate cancer cell migration and enhance radiation resistance by mediating intercellular communication. Importantly, EVs have significant impacts on the viability of neighboring tumor cells through radiation-induced bystander effects, resulting in resistance to radiotherapy and presents a significant barrier to effective cancer treatment^64^. For instance, miR-93-5p in cancer-associated fibroblasts can be transferred to colon cancer cells through EVs, causing radiation resistance in colon cancer^65^. Recent research has shown that MSC-EVs are involved in regulating resistance to cancer radiotherapy (Figure 2A).

A study conducted by Wan et al. discovered that miR-34c inhibits radiotherapy resistance of nasopharyngeal carcinoma both *in vitro* and *in vivo* through the β-Catenin signaling pathway^66^. Specifically, the delivery of miR-34c by MSC-EVs significantly inhibited the proliferation, migration, and radiotherapy resistance of nasopharyngeal carcinoma cells. In addition, it has been reported that a novel systemic cancer therapy can be achieved through the combination of local radiotherapy and MSC-EVs^67^. This combination therapy significantly reduced the growth and spread of melanoma cells. MSC-EV may serve as a chemical signal to induce apoptosis in cancer cells, thereby enhancing their susceptibility to radiation and facilitating systemic responses.

### MSC-EVs in chemotherapy resistance

Chemotherapy is the most effective treatment for cancer. Platinum-based compounds, paclitaxel (PTX), cyclophosphamide, 5-fluorouracil (5-FU), and doxorubicin (DOX), have emerged as first-line chemotherapy agents for improving the prognosis of cancer patients, owing to their broad-spectrum activity, minimal adverse effects, and cost-effectiveness^68^. However, chemotherapy resistance frequently emerges in clinical contexts, posing a significant obstacle in the field of cancer therapy. Consequently, it is imperative to conduct thorough investigations into the fundamental mechanisms that contribute to chemotherapy resistance in order to develop more efficacious approaches for treating cancer. A mounting body of research has established a robust association between MSC-EVs and chemotherapy resistance, due to the ability of MSC-EVs to transport biomacromolecules, such as lipids, functional proteins, and RNA, among others (Table 1 and Figure 2B). MSC-EVs have been demonstrated to regulate chemotherapy resistance in cancer, thereby affecting the efficacy of treatment and patient outcomes.

Research indicates that MSC-EVs can enhance cancer cell resistance to chemotherapy drugs through multiple pathways. The primary mechanism through which MSC-EVs enhance chemoresistance in tumor cells is by inhibiting apoptosis. miR-301b-3p delivered by UC-MSC-EVs was found to inhibit apoptosis in cisplatin (DDP)/vincristine-resistant gastric cancer cells, resulting in enhanced proliferation and migration, as well as heightened drug resistance, both *in vitro* and *in vivo*^69^. In another separate study, it has been demonstrated that UC-MSC-EVs exhibit the ability to augment the resistance of gastric cancer cells to the chemotherapeutic agents 5-FU and DDP^70^. Mechanistically, MSC-EVs stimulate the CaM-Ks/Raf/MEK/ERK pathway through their bioactive cargo, while concurrently increasing the expression of multidrug resistance-associated proteins, which counteracts apoptosis provoked by 5-FU and contributes to drug resistance. In addition, within tumor tissues, a distinct population of cells known as cancer stem cells (CSCs) possess the unique capacity for self-renewal and differentiation into various cell lineages^71^. In comparison to other tumor cells, CSCs demonstrate superior adaptability to the nutritional, metabolic, and hypoxic conditions of the TME, resulting in a higher likelihood of developing resistance to therapy. In the recent study by Lyu et al., it was demonstrated that BMSC-EVs increased the stemness of leukemia cells, resulting in leukemia metastasis^72^. In particular, BMSC-EVs enhance resistance to cytarabine treatment in leukemia by upregulating the expression of S100A4, a calcium-binding protein associated with malignant characteristics of cancers.

Moreover, under certain conditions, cancer cells undergo reversible cell cycle arrest, known as cancer dormancy, to adapt to stress and thrive in unfavorable surroundings^73^. Cancer dormancy is believed to contribute to cancer recurrence and the response to therapy. There is significant evidence indicating the presence of dormant breast cancer cells in the bone marrow, which contributes to breast cancer recurrence, metastasis, and drug resistance^34^, ^74^. A recent study discovered that breast cancer cells present in the bone marrow can trigger the secretion of miR-222/223 by MSCs via EVs^75^. This, in turn, leads to the prolonged immobility of dormant cells and resistance to carboplatin treatment. A similar scenario arose where miR-23b, transmitted by BMSC-EV, induced quiescence in breast cancer cells, inhibiting cellular viability and metastasis while promoting docetaxel resistance^76^. These findings provide a new perspective on cancer treatment and the reversal of drug resistance.

On the other hand, certain types of MSC-EVs have been demonstrated to counteract drug resistance in cancer cells and enhance their sensitivity to drugs, leading to improved therapeutic outcomes. In a study conducted by Luo et al., it was discovered that adipose-derived MSC (AMSC)-secreted EVs containing miR-122 can effectively enhance the sensitivity of HCC cells to 5-FU and sorafenib, which was achieved through the stimulation of cell apoptosis and cell cycle arrest^50^. Similar findings were observed in breast cancer. MiR-1236, carried by AMSC-EVs, can promote the inhibition of breast cancer cell viability and apoptosis induced by DDP by suppressing SLC9A1 and the Wnt/β-Catenin signaling pathway^77^. This, in turn, sensitizes breast cancer cells to DDP. DDP combined with AMSC-EVs may be a promising approach to enhance the effectiveness of breast cancer treatment. Additionally, the significance of EMT in promoting cancer metastasis and drug resistance has gained greater recognition^78^. In heterogeneous cancer cell populations, cells exhibiting the EMT phenotype demonstrate greater growth advantages in the presence of drugs. Cancer cells that are forcibly induced to activate the EMT program exhibit stem-like properties and, therefore, share signaling pathways and drug resistance traits with CSCs, manifested by increased drug efflux and anti-apoptotic effects^79^. Therefore, targeting EMT represents a promising strategy for addressing cancer resistance, offering a new opportunity in cancer therapy. Notably, miR-124 carried by BMSCs-EVs was able to inhibit proliferation and EMT, thereby increasing sensitivity to 5-FU treatment in pancreatic cancer cells^52^. However, further clarification is required regarding the precise mechanism of miR-124 carried by BMSC-EVs in regulating EMT and chemoresistance.

### MSC-EVs in targeted therapy resistance

With advancements in molecular biology and cell biology, precision therapy has become a predominant approach to cancer treatment. Targeted therapy is a type of precision treatment that uses drugs or other agents specifically created to impede the proliferation and dissemination of cancer cells by selectively targeting distinct molecular pathways and receptors within the tumor cells^80^. Several cancer-targeted drugs have been developed, including gefitinib for non-small cell lung cancer (NSCLC) with positive epidermal growth factor receptor (EGFR) expression, crizotinib for NSCLC with ALK mutations, sorafenib targeting VEGFR in various types of cancers, bevacizumab for metastatic colorectal cancer, and trastuzumab for breast cancer with HER-2 overexpression^68^. However, due to innate or acquired factors, targeted cancer therapy may also trigger drug resistance, ultimately leading to cancer recurrence and metastasis.

Recent research indicates that EVs released by MSCs can impact resistance to targeted cancer therapy (Table 2 and Figure 2C). Specifically, MSC-EVs have been observed to induce tolerance to targeted therapeutics in hematologic malignancies, thereby reducing the effectiveness of treatment. In chronic lymphocytic leukemia, MSC-EVs enhance cancer cell survival, migration, and resistance to several targeted drugs, including bortezomib (a proteasome inhibitor), ibrutinib (a Bruton tyrosine kinase inhibitor), idelalisib (a phosphoinositide-3 kinase inhibitor), and venetoclax (a Bcl-2 inhibitor)^81^. In addition, imatinib, a tyrosine kinase inhibitor, is currently the recommended initial treatment for chronic myeloid leukemia (CML)^68^. Nevertheless, the therapeutic efficacy of it is significantly impaired by drug resistance. In CML, MSC-EVs have been shown to inhibit cell proliferation and induce cell cycle arrest by transferring miR-15a^82^. Whereas in animal models, MSC-EVs appear to have a different impact, as they promote CML development and increase resistance to imatinib. Further research is needed to fully understand the involvement of MSC-EVs and their bioactive molecules in imatinib resistance in CML. Such analysis may potentially lead to new approaches and strategies for overcoming resistance to imatinib.

Of significant clinical relevance, the utilization of MSC-EVs can enhance the sensitivity of targeted drugs and yield superior therapeutic results in tumor treatment. Liu et al. reported that EVs derived from UC-MSCs enhanced the effectiveness of imatinib in promoting apoptosis and suppressing proliferation of CML cells via the caspase signaling pathway^77^. These findings indicate that incorporating MSC-EVs with targeted drugs may enhance the therapeutic efficacy of the drugs. Meanwhile, controlling MSC-EVs could serve as a pathway to alleviate resistance to targeted therapy in cancer.

### MSC-EVs in immunotherapy resistance

Cancer immunotherapy is a therapeutic strategy that stimulates the immune system to fight cancer^83^. Immunotherapy modifies the immune response of tumors, resulting in a reduced off-target impact compared to methods such as chemotherapy, which directly kill cancer cells.

Extensive research in tumor immunology, cell biology, and molecular technology has revealed that the TME plays a critical role in the development, metastasis, and immunosuppression of cancer^84^. Emerging evidence has demonstrated that EVs play a crucial role in regulating the proliferation, differentiation, polarization, and functioning of various immune cells within the TME. Significantly, MSC-EVs possess the capacity to modulate the differentiation and activity of diverse immune cell populations, including T cells, natural killer (NK) cells, macrophages, DCs, and B cells. (Figure 2D). For example, MSC-EVs suppress the proliferation, activation, and cytotoxicity of NK cells via the TGF-β/Smad2/3 signaling pathway^85^. MSC-EVs also affect the growth and function of T cells. EVs originating from IDO-1-overexpressed BMSCs have been shown to stimulate the growth of regulatory T cells (Tregs) while suppressing the proliferation of CD8^+^ T cells^86^. At the same time, they also inhibited the secretion of pro-inflammatory cytokines while augmenting the synthesis of anti-inflammatory cytokines. In addition, MSC-EVs can inhibit the activity of M1 macrophages and promote their polarization to M2 macrophages through the miR-let-7/IGF2BP1/PTEN signaling^87^.

Various approaches to cancer immunotherapy are commonly employed, including the utilization of immune checkpoint inhibitors that specifically target molecules involved in immune regulation, such as programmed cell death protein-1/ligand 1 (PD-1/PD-L1) and cytotoxic T lymphocyte-associated antigen-4, as well as chimeric antigen receptor T cell therapy^83^. Cancer immunotherapy has led to a long-lasting response in the majority of patients and has opened up new possibilities for advanced cancer therapies. Immunotherapy has had a significant impact on the treatment of diverse solid and hematologic malignancies, changing the therapeutic landscape. Checkpoint inhibitors have been authorized for the treatment of a wide range of malignant tumors, encompassing small and non-small cell lung cancer, gastric cancer, melanoma, renal cancer, head and neck carcinoma, and Hodgkin's lymphoma, yielding remarkable clinical outcomes^88^. Despite advances in cancer immunotherapy, some individuals may remain unresponsive due to primary, adaptive, or acquired resistance. Importantly, ongoing studies indicate that MSC-EVs have an effect on immune cells, which in turn impacts the response to cancer immunotherapy. MSC-EVs have been shown to induce the differentiation of monocytic myeloid-derived suppressor cells into immunosuppressive M2-polarized macrophages, thereby accelerating the progression of breast cancer^89^. Mechanistically, MSC-EVs contain cytokines such as TGF-β and semaphorins, which can lead to the overexpression of PD-L1 and the differentiation of MHC II-positive macrophages, ultimately promoting bone marrow tolerance. Further research is necessary to determine whether MSC-EVs and their bioactive substances have an inhibitory or promoting effect on tumor immunity. Furthermore, there is a need for further investigation to elucidate the impact of MSC-EVs on the resistance of cancer immunotherapy.

### MSC-EVs in endocrinotherapy resistance

Endocrinotherapy refers to the modification of the body's endocrine system in order to induce regression of hormone-related cancers, such as breast cancer, prostate cancer, thyroid cancer, ovarian cancer, and endometrial cancer^90^. Breast cancer and prostate cancer are the prevailing malignancies affecting women and men, respectively, with the highest incidence rates among all cancer types. In both diseases, the majority of cases exhibit hormone receptors that may be responsive to endocrinotherapy. However, some cancer patients may develop endocrine resistance due to both intrinsic and acquired factors. In breast cancer, for example, the altered interaction between the endoplasmic reticulum and growth factor receptors affects the response of cancer cells to endocrine treatment. Elevated expression of EGFR or HER2 has been demonstrated to impede the effectiveness of tamoxifen treatment in estrogen receptor-positive breast cancer^91^, ^92^. In addition, interactions between tumor cells and components of the TME, such as cancer-associated fibroblasts, immune cells, extracellular matrix, cytokines, and MSC-EVs, can contribute to cancer resistance to endocrinotherapy (Figure 2E). EVs from tamoxifen-resistant MCF-7 cells were found to promote tamoxifen resistance in recipient cells by selectively targeting P27 and ERα through miR-221/222^93^. Moreover, EVs contribute to breast cancer resistance to endocrine therapies by transporting various cargoes, including circRNAs, lncRNAs, and transfer RNA fragments^94^-^96^.

In prostate cancer, endocrinotherapy is typically achieved by either reducing or suppressing the production of androgens or inhibiting their activity within the body, commonly referred to as androgen deprivation therapy (ADT)^97^. This may involve procedures such as bilateral orchiectomy, the use of GnRH agonists or antagonists, estrogen therapy, or antiandrogen medication. However, prostate cancer patients who undergo ADT have a high risk of relapsing within 18 to 24 months and developing an aggressive form of the disease called metastatic castration-resistant prostate cancer (mCRPC), which, unfortunately, remains incurable^98^. Notably, EVs are also associated with the development of resistance to endocrine therapy in prostate cancer. Gan and co-authors discovered that miR-375 promotes the growth, migration, invasion, and resistance to the androgen receptor inhibitor enzalutamide in prostate cancer through the PTPN4/STAT3 signaling pathway^99^. Moreover, UC-MSC-EVs that containing miR-375 siRNA were observed to restrain the proliferation of mCRPC cells. In another separate study, the expression of miR-let-7c was down-regulated in mCRPC^100^. MSC-EV encapsulated miR-let-7c effectively decreased the proliferation and migration of mCRPC cells. Further investigations are necessary to elucidate the correlation between MSC-EVs and the cargo they contain, and their roles in facilitating resistance to cancer endocrinotherapy.

## Clinical Applications of MSV-EVs in Cancer Therapy Resistance

A deeper comprehension of the biological functions of MSC-EVs will enable us to discover more efficient strategies to reduce or even reverse resistance to cancer therapy, ultimately improving the therapeutic efficacy of cancer treatment. In addition, drug and biomolecular delivery systems have been developed using MSC-EVs, and researchers are currently investigating the potential of MSC-EVs in reducing the adverse effects of cancer therapy.

### MSC-EVs enhance chemosensitivity in cancer

Currently, the effectiveness of chemotherapy in treating solid tumors, which constitute over 90% of malignant tumors, remains unsatisfactory. Resistance of cancer cells to chemotherapy drugs is a significant factor contributing to treatment failure, and addressing this issue is a pressing chemotherapy concern. A large number of studies have confirmed the important role of MSC-EVs in cancer drug therapy resistance, suggesting their potential in reversing chemotherapy resistance and improving drug sensitivity (Figure 2B). HCC is a malignant growth that is a widespread concern worldwide and contributes significantly to cancer-related mortality. The effectiveness of chemotherapy in treating HCC is hindered by the development of resistance, which negatively affects patient outcomes. Notably, recent research suggests that MSC-EVs may hold the potential to improve or even reverse chemotherapy resistance in HCC. For example, Lou et al. discovered that AMSCs selectively package miR-122 into EVs and transfer them to HCC cells, leading to increased apoptosis and cell cycle arrest in HCC cells^50^. Pharmacological studies have indicated that miR-122, delivered by AMSC-EVs, modulates the expression of targeted genes in HCC, including CCNG1, ADAM10, and IGF1R, subsequently enhances sorafenib sensitivity and the anti-tumor effectiveness of HCC, both *in vitro* and *in vivo*. Similarly, AMSC-EVs facilitate the transfer of miR-199a to HCC, thereby enhancing the sensitivity of HCC cells to the chemotherapy agent DOX by regulating the mTOR pathway, both in cellular and animal models^101^. In another study, Xu et al. demonstrated that UC-MSCs could upregulate miR-451a expression, which subsequently downregulates ADAM10 in HCC, thereby promoting apoptosis and inhibiting proliferation and suppressing PTX resistance in HCC cells^102^. Additionally, glucose-regulated protein 78 (Grp78) has been strongly implicated in the pathogenesis of cancer. Li et al. demonstrated that Grp78 is associated with the progression of HCC and resistance to sorafenib, suggesting that targeting the expression of Grp78 could be an effective strategy for inhibiting the development of HCC and overcoming chemotherapy resistance^73^. Delivering Grp78 siRNA through BMSC-EVs enhances the sensitivity of HCC cells to sorafenib and reverses their resistance to the drug. By adopting MSC-EVs, innovative strategies and application paradigms can be developed to enhance the effectiveness of chemotherapy for HCC.

MSC-EVs have a facilitation on the chemosensitivity of various types of cancer. In breast cancer, AMSC-EVs can suppress the expression of SLC9A1 through miR-1236, resulting in the inhibition of the Wnt/β-catenin signaling pathway and thereby enhancing the sensitivity of breast cancer cells to DDP^103^. In addition, it was confirmed that BMSC-EVs-delivered miR-193a inhibits the growth, motility, and invasiveness of DDP-resistant NSCLC cells^104^. Meanwhile, it stimulates apoptosis by targeting LRRC1. Moreover, miR-124, which is transported by MSC-EVs, exerts an inhibitory effect on glioblastoma (GBM) migration by targeting CDK6, while also enhancing the susceptibility of GBM cells to temozolomide (TMZ) therapy^105^. Another separate study found abnormal expression of miR-9 in TMZ-resistant GBM, suggesting that inhibiting miR-9 could potentially reverse TMZ resistance^82^. Subsequent studies have shown that delivering anti-miR-9 to GBM cells through MSC-EVs leads to a reduction in the expression of multidrug transporters, which, in turn, makes GBM cells more sensitive to TMZ and greatly improves the effectiveness of anti-cancer treatment. These results indicate that utilizing MSC-EVs could be an effective strategy for enhancing outcomes in cancer chemotherapy.

### MSC-EVs expand the efficacy of cancer immunotherapy

The effectiveness of immunotherapy in the clinical management of various malignancies has been firmly established. It has shown promise in producing positive results even in advanced cancer cases, including those that do not respond to standard therapies. To overcome the limitations of immunotherapy, including mutation targeting, metabolic pathway heterogeneity, immunosuppression, technical challenges, and high costs, clinicians often utilize a combination of other therapies alongside immunotherapy in clinical practice. This approach includes the use of chemoimmunotherapy (CIT) and other methods to enhance the therapy efficacy, offering new perspectives for cancer treatment^106^. Studies have demonstrated the use of CIT in preclinical studies and clinical trials for melanoma, lung cancer, breast cancer, and liver cancer. Past that, MSC-EVs present great potential (Figure 2D). In a recent study of Tan's group, they utilized MSCs to produce anti-CD3/CD20 bispecific antibodies and EVs for the treatment of B-cell lymphoma^107^. Mechanistically, MSC-EVs were able to carry miR-15a/miR-16, which resulted in downregulation of BCL-2 expression and subsequent induction of apoptosis. Meanwhile, anti-CD3/CD20 bispecific antibodies secreted by MSCs activate cytotoxic T lymphocytes and facilitate the release of TNF-α and interferon-γ, thereby synergistically combating B-cell lymphoma. Further, Zhou et al. engineered MSC-EVs modified with an oxaliplatin prodrug and loaded with siRNA targeting galectin-9^108^. This system promotes anti-tumor immunity by inducing macrophage polarization, enhancing recruitment of cytotoxic T lymphocytes, and reducing Tregs, thus improving the efficacy of anti-tumor therapy in pancreatic cancer. However, the impact of chemotherapy drugs on immunotherapy is debated. Delineating the fundamental mechanisms by which MSC-EVs modulate cancer immunotherapy and enhance its translational utility can provide new insights into addressing various types of cancer, particularly those that display drug-resistant characteristics.

### MSC-EVs as carriers for anti-cancer materials delivery

Another important clinical application of EVs is their ability to transport functional materials such as RNAs, proteins, and drugs to specific target cells. Compared to other conventional carriers such as liposomes, EVs have distinct advantages in terms of targeting specificity, low immunogenicity, permeability across biological barriers, and flexibility of modification^7^. Specifically, EVs can enter cancer cells through transcytosis and macropinocytosis pathways to release chemotherapy drugs, leading to enhanced drug accumulation deep inside cells and avoiding excretion by drug efflux pumps in the cell membrane such as ATP-binding cassette transporters^109^-^111^. This mechanism allows for increased intracellular drug accumulation, leading to improved therapeutic outcomes at lower dosages.

There are two modes of cargo loading depending on whether EVs are purified before or after the cargo is loaded: pre-loading and post-loading^112^. Pre-loading refers to the process of loading cargo during EV biogenesis in order to generate specific products. This method enables the fusion of desired cargoes, such as proteins and short peptides, with markers on the surface of EVs, or facilitates the encapsulation of cargo during EV formation. The post-loading technique enables the direct loading of cargoes into the EV after extraction. Research has indicated that loading approaches can affect encapsulation efficiency, the integrity of electric vehicles, and cell uptake. For instance, DOX can be loaded into EVs through various techniques such as co-incubation, electroporation, extrusion, freeze-thawing, sonication, and surfactant treatment^113^. Considering factors such as encapsulation efficiency, particle concentration, and loading capacity, co-incubation and electroporation are currently the optimal strategies for loading DOX into EVs. Numerous natural and engineered EVs have been considered as ideal delivery systems for anti-cancer materials (Table 3).

As previously stated, MSCs have the ability to release EVs that contain specific types of RNAs, including ncRNAs, siRNAs, mRNAs and others^8^. After internalization, EVs carry RNAs that can regulate cancer progression and response to therapy. MiRNAs have emerged as key players in MSC-EV delivery research. The delivery of miRNAs through MSC-EVs primarily involves two approaches: miRNA substitution and miRNA inhibition^114^. The former involves the introduction of exogenous miRNAs or miRNA mimics that possess inhibitory effects on cancer cells. On the other hand, the latter approach employs specific miRNA inhibitors or antisense oligonucleotides to suppress cancer-promoting miRNAs. Numerous studies have reported a growing number of successful instances in this regard. MSC-EVs have the ability to deliver a range of anti-cancer miRNAs, such as miR-16-5p, miR-34a, miR-101, miR-124, and miR-499a-5p, which can potentially act as a therapeutic approach in combating cancer^10^, ^105^, ^115^-^117^. In a recent study by Qin et al., miR-208a was found to enhance the proliferation, migration, and invasion of osteosarcoma cells^118^. Further, they employed BMSC-EVs as carriers for miR-208a inhibitor (anti-miR-208a), which possess substantial inhibition of cell viability, clonogenicity, and migration of osteosarcoma cells. These findings offer promising prospects for the development of novel therapeutic strategies for osteosarcoma. Furthermore, various RNA species, including mRNAs, siRNAs, and lncRNAs, have been incorporated into the delivery strategies of MSC-EVs for anti-cancer purposes^8^, ^119^-^121^. Ongoing research in this area is yielding promising results, and the successful delivery of these RNA molecules via MSC-EVs holds the potential to inform future approaches to cancer therapy.

Proteins are the primary agents of life. The researchers load tumor necrosis factors, enzymes, and other functional proteins into MSC-EVs to investigate their targeted delivery capabilities and potential applications in cancer therapy. In a recent study by Zhuang and colleagues, MSC-EVs modified with superparamagnetic iron oxide nanoparticles (SPIONs) were engineered to deliver TNF-α to melanoma cells^122^. The apoptotic pathway was activated, resulting in the inhibition of cancer cell growth. The TNF-α-EV-SPIONs nanoparticles exhibit enhanced targeting capabilities and reduced toxicity, offering promising anti-cancer effects. In addition, Yuan et al. constructed MSC-EVs that expressed TNF-related apoptosis-inducing ligand (TRAIL) on their surfaces, resulting in the apoptosis of various cancer cells upon internalization and overcoming TRAIL resistance^123^. Since MSC-EV delivery can result in protein overexpression within cells, it is unclear whether this affects any other functions mediated by proteins. Further investigation is needed to assess the clinical efficacy of using MSC-EVs for protein delivery in cancer therapy.

Beyond that, MSC-EVs loaded with small molecule drugs, such as DOX, PTX, 5-FU, and DDP, have been investigated for a long time, with some undergoing clinical trials^124^-^127^. Drugs are gradually released from EVs and continuously exert their effects on targeted cells, resulting in increased drug concentration within the targeted cells and a prolonged circulation time in the bloodstream. As a result, it enhances anti-tumor efficacy. DOX is a chemotherapeutic agent that is commonly used as a first-line treatment due to its strong anti-tumor properties. However, its therapeutic efficacy is hindered by the occurrence of significant adverse reactions, especially cardiotoxicity. Wei et al. investigated the potential of using BMSC-EVs as DOX carriers in the treatment of osteosarcoma^128^. They demonstrated that DOX delivered by BMSC-EVs had a more pronounced anti-osteosarcoma effect compared to free DOX. Notably, BMSC-EV-mediated delivery of DOX exhibits greater accumulation in tumor tissue than organs such as the heart, demonstrating enhanced tumor targeting efficacy and reduced potential for toxicities and adverse effects. In addition, given the high clinical use of PTX, there are also instances of adverse reactions. Therefore, it is imperative to reduce the dosage of PTX and improve its treatment efficiency simultaneously. Research has found that mouse MSCs can be used to encapsulate PTX, leading to the formation of PTX-containing EVs, which exhibit a pronounced inhibitory effect on the proliferation of pancreatic cancer cells *in vitro*^129^. In another separate study by Zhou et al., the antitumor effects of BMSC-EVs loaded with PTX and gemcitabine against pancreatic cancer were found to be significantly enhanced, which can be attributed to inducement of apoptosis and inhibition of the cell cycle^126^. Moreover, Melzer et al. discovered that MSC-EVs loaded with PTX exhibited improved therapeutic efficacy against breast cancer, inhibiting the growth of primary and metastatic tumors at lower concentrations both *in vivo* and *in vitro*^127^. Similarly, Abas and colleagues found that active loading of PTX into MSC-EVs resulted in induction of apoptosis and inhibition of cell migration in cervical cancer, even at lower concentrations^130^. These results suggest that by using MSC-EVs as a delivery system for PTX, not only can biological barriers be overcome and chemotherapy resistance be circumvented, but also the potential for mild systemic toxicity is observed, holding promise as a therapeutic strategy for cancer.

A growing body of evidence indicates that natural products and their derivatives derived from traditional Chinese medicine exhibit promising anti-tumor properties. Liang and colleagues use BMSC-EVs as a carrier for Norcantharidin (NCTD), a natural anti-tumor agent with increasing leukocyte properties, for the treatment of HCC^131^. The delivery of NCTD by BMSC-EVs enhances cytotoxicity compared to free NCTD, and effectively inhibits HCC cell migration and invasion, while promoting apoptosis. These findings provide novel insights and potential therapeutic strategies for the management of HCC. Additionally, Honokiol, a phytochemical with broad anti-tumor properties, faces challenges in its clinical use due to its limited cell uptake rate and bioavailability. To address this problem, Kanchanapally et al. used BMSC-EVs that were modified with surface markers to deliver Honokiol, which successfully increased Honokiol accumulation in HCC cells and induced cell cycle arrest and apoptosis^131^. Collectively, these investigations provide evidence that the delivery of antitumor active substances via MSC-EVs can enhance precision in targeting and uptake of drugs, leading to enhanced therapeutic outcomes with reduced drug dosages.

### Assessment of MSC-EVs in cancer clinical trials is ongoing

At present, the clinical trial database (https://www.clinicaltrials.gov) encompasses a comprehensive collection of more than 150 entries pertaining to the examination of interactions between EVs and a range of diseases, notably pneumonia, sepsis, cerebral hemorrhage, COVID-19, preeclampsia, and particularly cancer. These entries predominantly focus on diverse aspects such as the identification of biomarkers for diagnostic and prognostic evaluation, therapeutic interventions, drug delivery mechanisms, and the development of cancer vaccines. EVs have received extensive attention in both preclinical and clinical studies as a promising biological system for drug delivery^132^, ^133^. By loading drugs or genes into EVs and performing surface modifications, precise targeting of specific sites can be achieved. There are currently several clinical trials registered on the clinical trial database for the use of MSC-EVs in cancer therapy.

KRAS mutations are prevalent in various types of cancers and adenocarcinomas, with G12D mutations being the most frequently observed^134^. Extensive research has been conducted at different levels, including molecular, cellular, organoid, and animal models, to investigate the structure and conformation of KRAS^G12D^, as well as its associated signaling pathways (such as PI3K/AKT signaling), omics information, and targeted inhibitors, and information has been obtained on cancer malignancy phenotype, behavior, and therapeutic response. The KRAS^G12D^ mutation demonstrates significant oncogenic capacity in a variety of *in vitro* and *in vivo* models^135^-^137^. In addition, KRAS^G12D^ mutations have been found to be often associated with unfavorable overall survival outcomes in a significant proportion of cancer patients and exhibit resistance to anti-EGFR and anti-PD-1 therapy^138^-^140^. In terms of KRAS^G12D^ inhibitors, numerous preclinical and clinical investigations have been conducted, such as MRTX1133, RMC-9805, TH-Z835, HRS-4642 (NCT05533463), TH-Z835 (NCT05382559), and others^141^, ^142^. Specifically, in a Phase Ⅰ trial, the researchers produced MSC-EVs that transmit siRNA targeting KRAS^G12D^ to treat metastatic pancreatic cancer carrying the KRAS^G12D^ mutation (ClinicalTrials.gov Identifier: NCT03608631). The utilization of MSC-EVs as a vehicle to deliver KRAS^G12D^ inhibitors has the potential to provide novel insights into targeted cancer therapy, ultimately leading to enhanced patient outcomes. The use of MSC-EVs in the delivery of antineoplastic drugs offers a potential opportunity for cancer treatment. Despite promising outcomes in the utilization of MSC-EVs as vehicles in clinical cancer therapy, several obstacles persist. Improvements and refinements are required in the MSC-EV standard purification protocol and production process. In addition, to ensure the biological safety of a treatment, it is necessary to investigate the interrelationship between the administration route, drug dosage, and pharmacokinetics.

## Challenges and Perspectives

### Current limitations of MSC-EVs in cancer

Over the past few decades, research on EVs has made significant progress, with a particular focus on investigating their potential to improve human health. MSC-EVs play a crucial role in various types of malignant tumors. These EVs contain a diverse array of bioactive cargoes that have the potential to serve as novel targets for overcoming drug resistance in cancer therapy. Specifically, the use of MSC-EVs shows promising potential for clinical cancer treatment, as they act as efficient carriers for delivering anti-cancer drugs and biologics. Despite valuable research, there are still challenges and limitations that hinder the widespread adoption of MSC-EVs.

First, given the complex and heterogeneous nature of MSC-EVs in terms of their composition and functions, it is imperative to develop standardized protocols for the nomenclature, classification, isolation, and characterization of EVs prior to their use in experimental and clinical settings. Second, safety is a crucial concern in the clinical implementation of MSC-EVs. The function of MSC-EVs in cancer treatment and drug resistance is complex and context-dependent, influenced by factors such as the source of MSCs and the specific cancer being targeted^143^. Numerous research has documented the tumor-promoting properties of EVs derived from MSCs, thereby impeding their potential clinical utility, especially in cancer patients. Notably, MSC-derived EVs have been shown to stimulate cancer cell proliferation, inhibit apoptosis, and facilitate intratumoral angiogenesis through miRNAs transfer^144^-^146^. Third, enhancing the efficient bioavailability of EVs is a matter that requires careful consideration. Although several studies have demonstrated the tumor-homing properties of MSC-EVs, they are inevitably internalized by other cells as well. In addition, several groups have demonstrated that intravenous administration of MSC-EV results in prompt clearance by macrophages of the monocyte phagocyte system (MPS) from the bloodstream, and subsequently tend to accumulate in MPS organs, including the liver, spleen, and lungs, thereby significantly limiting their biodistribution and bioavailability to tumor tissues^110^, ^111^, ^147^. Fourth, the yield of natural MSC-EVs and the efficacy of their drug delivery are generally low, which hinders their extensive investigation and clinical implementation. BMSCs, UC-MSCs, and AMSCs are currently primarily utilized for the production of EVs. Nevertheless, obtaining samples for extracting is a challenging task, resulting in limited quantities of EVs.

### Future Opportunities in MSC-EVs Research

In order to effectively tackle the challenges and barriers faced in the investigation and medical application of MSC-EVs in cancer, it is imperative for future studies to prioritize the following areas. In order to mitigate the impact of inconsistent isolation and purification techniques, it is imperative to establish uniform international standards for the isolation and characterization of MSC-EVs. Future research should also focus on characterizing and differentiating subpopulations of MSC-EVs obtained from diverse sources. Based on this, the comprehensive investigation of MSC-EVs and their cargoes in the potential of treatment, monitoring drug resistance, and evaluating prognosis provides a new perspective for fully understanding the biological mechanisms underlying cancer. This, in turn, offers novel strategies for the clinical prevention and management of cancer.

To ensure the safety and bioavailability of MSC-EVs for clinical use, it is essential to utilize engineering strategies to eliminate or neutralize any unwanted or harmful substances present within them. Recent developments in the fields of nanotechnology and biomedical engineering hold promise in addressing the issue of limited production of MSC-EVs and, significantly, facilitating the precise targeting of MSC-EVs towards cancer cells (Figure 1C). Over the past few years, researches have been focused on developing engineered EVs to function as excellent drug delivery systems. Engineered EVs share similar characteristics and applications as natural EVs, while offering the potential for personalized customization and mass production. Importantly, scientists have applied surface engineering technology to natural MSC-EVs in an effort to attach guiding peptides, biological groups, and chemical groups to their surfaces. For instance, Zhuang et al. developed a synergistic system involving fusion proteins of cell-penetrating peptides (CPP) and TNF-α anchored EVs, which were further coupled with SPIONs^122^. The incorporation of CPP significantly enhanced the binding of TNF-α to its receptor, while SPIONs facilitated the localization of EVs to the tumor site under magnetic field, thereby improving the targeting efficiency. In addition, by surface modification, MSC-EV internalization by macrophages in the liver and spleen may be diminished, thereby extending their duration in circulation and enhancing their accumulation within tumors. For instance, in a combined strategy, cationized mannan-EVs derived from DC2.4 cells were employed to saturate MPS^148^. At the same time, EVs were coated with CD47 to prevent their phagocytosis by MPS. This integrated approach has the potential to extend the presence of MSC-EVs in the bloodstream and increase tumor distribution, thus enhancing their anti-tumor efficacy. Some other cellular surface markers, such as lysosomal-associated membrane protein 2, platelet-derived growth factor receptor, CD63, CD9, and CD81 can be used to engineer modifications that confer cell-selective targeting to MSC-EVs^149^.

Moreover, several studies have utilized MSC-EV mimics (MSC-EMs) as drug delivery vehicles, resulting in improved drug loading efficiency and enhanced anti-cancer treatment outcomes. For example, Wang et al. prepared DOX-coated EMs for the treatment of osteosarcoma by extruding them into BMSCs^150^. BMSC-EMs loaded with DOX exhibited superior anti-cancer efficacy and decreased adverse effects compared to free DOX. Similarly, in a study by Kalimuth's group, PTX-coated BMSC-EMs were produced using extrusion and ultracentrifugation. The EMs exhibited positivity for ALIX and CD63 markers and were of EV-like dimensions^151^. Functional investigations have indicated that the BMSC-EMs exhibit remarkable inhibition of breast cancer cell proliferation and could serve as a drug delivery system for anti-breast cancer therapy.

In the future, MSCs could potentially be obtained from easily accessible tissue sources such as menstrual blood and dental pulp, which are non-invasive^152^, ^153^. In addition, induced pluripotent stem cells (iPSCs) are a type of stem cell that exhibit the ability to self-renew and differentiate in a manner comparable to embryonic stem cells., which are generated from terminally differentiated cells through *in vitro* reprogramming technology^154^. Studies have demonstrated that iPSCs have the ability to produce a substantial quantity of MSCs that possess consistent biological characteristics, including continuous growth, the same tumor homing capacity, and much less risk of tumorigenesis. Using patient-specific iPSC-MSCs for autologous transplantation can avoid immune rejection and yield positive outcomes in regenerative medicine.

## Conclusion

MSCs are a subpopulation of multipotent precursor cells present in various tissues, capable of differentiating into diverse cell types^3^. Apart from their ability to self-renew, MSCs demonstrate direct or indirect interactions with a range of cells within the TME, influencing the development and advancement of cancer. Specifically, EVs, a critical constituent of the MSC-secretome, have an impact on cancer biology and the response to therapy by transferring bioactive molecules and cell signals with neighboring cells, providing a novel perspective on the clinical management of cancer^6^.

In this paper, we summarize the biological characteristics of MSC-EVs and their important role in cancer therapy resistance. Currently, with the large number of studies on MSC-EVs, it is evident that there are increasing opportunities to utilize them for regulating and reversing therapy resistance in cancer. Despite the recent prominence of MSC-EVs as biological delivery systems, it is still necessary to develop appropriate technologies in combination with engineering strategies to customize MSC-EVs with an enhanced drug loading capability, improved targeting specificity, and reduced cytotoxicity. Further studies should also be conducted at the animal and *in vivo* levels to provide direct evidence for clinical application. The understanding of MSC-EVs can be further expanded to capitalize on their potential in cancer therapy.

## Figures and Tables

**Table 1:** MSC-EVs in cancer chemotherapy resistance.

Cancer type	MSC source	Cargo carried	Target	Drug	Effect on drug resistance	Ref.
HCC	AMSC	miR-122	CCNG1/ADAM10/IGF1R	Sorafenib	Inhibit	50
	AMSC	miR-199a	mTOR	Doxorubicin	Inhibit	101
	BMSC	GRP78 siRNA	N.D.	Sorafenib	Inhibit	155
GC	UC-MSC	N.D.	CaM-Ks/Raf/MEK/ERK	5-Fluorouracil	Promote	70
	UC-MSC	miR-301b-3p	TXNIP	Cisplatin	Promote	69
BC	BMSC	miR-222/223	N.D.	Carboplatin	Inhibit	75
	AMSC	miR-1236	SLC9A1 Wnt/β-Catenin pathway	Cisplatin	Inhibit	103
	BMSC	miR-23b	MARCKS	Docetaxel	Promote	76
	BMSC	miR-21-5p	S100A6	Doxorubicin	Promote	49
Pancreatic cancer	BMSC	miR-124	EZH2	5-Fluorouracil/5-Fluorouracil plus	Inhibit	52
	AMSC	lncRNA NEAT1	miR-491-5p/Snail/SOCS3	Gemcitabine	Promote	121
AML	BMSC	miR-142-3p	S100A4	Cytarabine	Promote	72
Ovarian cancer	UC-MSC	miR-146a	LAMC2/PI3K/Akt	Docetaxel/Taxane	Inhibit	51
LC	BMSC	miR-193a	LRRC1	Cisplatin	Inhibit	104
Glioblastoma	MSC	anti-miR-9	MDR1	Temozolomide	Inhibit	156

AML: Acute myeloid leukemia; AMSC: Adipose-derived mesenchymal stem cell; BC: Breast cancer; BMSC: Bone marrow mesenchymal stem cell; LC: Lung cancer; GC: Gastric cancer; HCC: Hepatocellular carcinoma; UC-MSC: Umbilical cord mesenchymal stem cell.

**Table 2:** MSC-EVs in cancer targeted therapy resistance.

Cancer type	MSC source	Cargo carried	Target	Drug	Effect on drug resistance	Ref.
AML	BMSC	TGFB1, miR-155 and miR-375	N.D.	Cytarabine AC220	Promote	157
CLL	BMSC	N.D.	CCL3/4, EGR1/2/3, and MYC	Fludarabine Ibrutinib Idelalisib Venetoclax	Promote	81
CML	BMSC	miR-15a	Bcl-2 and Caspase3	Imatinib	Promote	82
	UC-MSC	N.D.	Caspase pathway	Imatinib	Inhibit	77
MM	MSC	lncPSMA3-AS1	PSMA3	Bortezomib Carfilzomib	Promote	158

AML: Acute myeloid leukemia; BMSC: Bone marrow mesenchymal stem cell; CLL: Chronic lymphocytic leukemia; CML: Chronic myelogenous leukemia; MM: Multiple myeloma; MSC: Mesenchymal stem cell; UC-MSC: Umbilical cord mesenchymal stem cell.

**Table 3:** Delivery of miRNAs, proteins and drugs by MSC-EVs.

Cancer Type	RNAs, proteins and drug	MSC source	Loading methods	Anti-cancer effect	Ref.
Glioma	miR-124	BMSC	Transfection	Inhibit cell migration, enhance the to temozolomide	105
	miR-146b	MSC	Electroporation	Inhibit cell growth	159
	miR-584-5p	MSC	Lentivirus infection	Inhibit cell proliferation and migration	160
	CDA/miR-34a	UC-MSC	Transfection	Synergistically promote cell apoptosis	161
Pancreatic cancer	miR-124	BMSC	Transfection	Inhibit cell growth	52
	lncRNA NEAT1	AMSC	Transfection	Promote proliferation, migration, and gemcitabine resistance	121
	Galectin-9 siRNA/Oxaliplatin	BMSC	Electroporation for siRNA, incubation for Oxaliplatin	Induce anti-tumor immunity, increase the accumulation in tumor	108
	KRAS^G12D^ siRNA	UC-MSC	Electroporation	inhibit proliferation, viability and migration, enhance apoptosis	119
	Paclitaxel/GEMP	BMSC	Electroporation	Inhibit cell growth	126
	Paclitaxel	BMSC	Incubation	Inhibit cell growth	129
Breast cancer	miR-16-5p	AMSC	Transfection	Inhibit proliferation and migration, enhance apoptosis	115
	miR-34a	DPSCs	Lentivirus infection	Inhibit cell growth, migration, and invasion	116
	miR-142-3p	BMSC	Electroporation	Inhibit cell growth, induce apoptosis	162
	miR-145	AMSC	Lentivirus infection	Inhibit apoptosis and metastasis	163
	miR-379	BMSC	Lentivirus infection	Inhibit cell growth and migration	164
	miR-381-3p	AMSC	Incubation	Inhibit proliferation, migration, invasion and EMT	165
	Paclitaxel	BMSC	Incubation	Inhibit cell growth	151
	Doxorubicin	MSC	Electroporation	Promote cell uptake	166
HCC	miR-122	AMSC	Transfection	Inhibit cell growth, enhance sensitivity to sorafenib	50
	miR-199a	AMSC	Lentivirus infection	Increase the toxic effect of Doxorubicin	101
	Doxorubicin	MSC	Incubation	Inhibit proliferation and migration, promote apoptosis	167
	Norcantharidin	BMSC	Electroporation	Induce cell cycle arrest, inhibit proliferation, promote apoptosis	131
Osteosarcoma	miR-101	AMSC	Lentivirus infection	Inhibit invasion and migration *in vitro*, inhibit metastasis *in vivo*	10
	Doxorubicin	MSC	Incubation	Inhibit cell growth	128
Prostate cancer	miR-let-7c	BMSC	Transfection	Inhibit proliferation and migration	100
Lung cancer	miR-21-5p	BMSC	Transfection	Promote proliferation, survival, invasiveness, EMT and macrophage M2 polarization	145
Neuroblastoma	miR-124	AMSC	Transfection	Inhibit proliferation and promote differentiation.	168
Endometrial cancer	miR-499a-5p	BMSC	Electroporation	Inhibit cell growth and metastasis	117
Bladder cancer	PLK-1 siRNA	MSC	Electroporation	Inhibit cell growth	120
Melanoma	TNF-α	MSC	Transfection	Promote apoptosis and inhibit cell growth	122
Gastric cancer	L-PGDS	UC-MSC	Adenovirus infection	Inhibit cell growth, migration, and invasion, induce apoptosis	169
Breast cancer Colorectal cancer Kidney carcinoma Lung cancer	TRAIL/PTEN/IFN-β1	AMSC	Lentivirus infection	Active immune cells and induce apoptosis	170
Lymphoma	Anti-CD3/CD20 BsAb	UC-MSC	Transfection	Inhibit cell growth	107
Cervical cancer	Paclitaxel	UC-MSC	Electroporation	Induce apoptosis, inhibit EMT	130
Oral carcinoma	CTX/TRAIL	MSC	Incubation for CTX, transfection for TRAIL	Synergistically inhibit cancer cell growth and neoplasia	171
Thyroid cancer	New TKI^a^	AMSC	Incubation	Promote sensitivity to radiotherapy	172
Colorectal cancer	Doxorubicin	BMSC	Electroporation	Inhibit cell growth, increase liver clearance	124
Lung cancer Ovarian cancer Breast cancer	Taxol	MSC	Incubation	Inhibit cell growth, reduce the accumulation in organs	127
Pancreatic cancer Breast cancer Colon cancer Prostate cancer Ovarian cancer	Honokiol	MSC	Sonication	Induce cell cycle arrest and apoptosis, inhibit cell growth	173

AMSC: Adipose-derived mesenchymal stem cell; BMSC: Bone marrow mesenchymal stem cell; BsAb: Bispecific antibodies; CDA: Cytosine deaminase; CTX: Cyclophosphamide; DPSC: Dental pulp-derived MSC; EMT: Epithelial to mesenchymal transition; GEMP: Gemcitabine monophosphate; L-PGDS: Lipocalin-type prostaglandin D2 synthase; MSC: Mesenchymal stem cell; New TKI^a^: 5-(5-{4H,5H,6H-cyclopenta [b]thiophen-2-yl}-1,3,4-oxadiazol-2-yl)-1-methyl-1,2-dihydropyridin-2-one; PLK-1: Polo-like kinase 1; UC-MSC: Umbilical cord mesenchymal stem cell.

## Abbreviations

5-FU: 5-fluorouracil
ADT: androgen deprivation therapy
ALIX: apoptosis-linked protein 2-interacting protein X
AMSC: adipose-derived mesenchymal stem cell
BMSC: bone marrow mesenchymal stem cell
circRNA: circular RNA
CIT: chemoimmunotherapy
CML: chronic myeloid leukemia
CPP: cell-penetrating peptides
CSC: cancer stem cell
DC: dendritic cell
DDP: cisplatin
DLS: dynamic light scattering
DOX: doxorubicin
EGFR: epidermal growth factor receptor
EM: electron microscopy
EMT: epithelial-mesenchymal transition
ESCRT: endosomal sorting complexes required for transport
ESE: early-sorting endosome
EV: extracellular vesicle
GBM: glioblastoma
Grp78: glucose-regulated protein 78
HCC: hepatocellular carcinoma
HSP: heat shock protein
ILV: intraluminal vesicle
iPSC: induced pluripotent stem cell
lncRNA: long noncoding RNA
LSE: late-sorting endosome
miRNA: microRNA
mCRPC: metastatic castration-resistant prostate cancer
MPS: monocyte phagocyte system
MSC: mesenchymal stem cell
MSC-EM: MSC-EV mimic
MVB: multi-bubble body
ncRNA: noncoding RNA
NCTD: norcantharidin
NK: natural killer
NSCLC: non-small cell lung cancer
NTA: nanoparticle tracking analysis
PD-1/PD-L1: programmed cell death protein-1/ligand 1
PEG: polyethylene glycol
PTX: paclitaxel
SEC: size exclusion chromatography
SPIONs: superparamagnetic iron oxide nanoparticles
TME: tumor microenvironment
TMZ: temozolomide
TRAIL: TNF-related apoptosis-inducing ligand
Treg: regulatory T cell
TSG101: tumor susceptibility gene 101
UC-MSC: human umbilical cord mesenchymal stem cell
